# Image quality assessment along the one metre axial field-of-view of the total-body Biograph Vision Quadra PET/CT system for ^18^F-FDG

**DOI:** 10.1186/s40658-022-00516-5

**Published:** 2022-12-14

**Authors:** Ivo Rausch, Julia G. Mannheim, Jürgen Kupferschläger, Christian la Fougère, Fabian P. Schmidt

**Affiliations:** 1 QIMP Team, Center for Medical Physics and Biomedical Engineering, Medical University of Vienna, Waehringer Guertel 18-20/4L, 1090 Vienna, Austria; 2 Werner Siemens Imaging Center, Department of Preclinical Imaging and Radiopharmacy, Eberhard-Karls University Tuebingen, Tübingen, Germany; 3 Cluster of Excellence iFIT (EXC 2180) “Image Guided and Functionally Instructed Tumor Therapies”, University of Tuebingen, Tübingen, Germany; 4 Department of Nuclear Medicine and Clinical Molecular Imaging, University Hospital Tuebingen, Otfried-Mueller-Strasse 14, 72076 Tübingen, Germany

**Keywords:** Total-body PET, Biograph Vision Quadra, Extended axial FOV, Image quality

## Abstract

**Aim:**

Recently, total-body PET/CT systems with an extended axial field-of-view (aFOV) became commercially available which allow acquiring physiologic information of multiple organs simultaneously. However, the nominal aFOV may clinically not be used effectively due to the inherently reduced sensitivity at the distal ends of the aFOV. The aim of this study was to assess the extent of the useful aFOV of the Biograph Vision Quadra PET/CT system.

**Methods:**

A NEMA image quality (IQ) phantom mimicking a standard [^18^F]FDG examination was used. Image contrast and noise were assessed across the 106 cm aFOV of the Biograph Vision Quadra PET/CT system (Siemens Healthineers). Phantom acquisitions were performed at different axial positions. PET data were rebinned to simulate different acquisition times for a standard injected activity and reconstructed using different filter settings to evaluate the noise and images along the axial direction.

**Results:**

Image noise and contrast were stable within the central 80 cm of the aFOV. Outside this central area, image contrast variability as well as image noise increased. This degradation of IQ was in particular evident for short acquisition times of less than 30 s. At 10 min acquisition time and in the absence of post-reconstruction filtering, the useful aFOV was 100 cm. For a 2 min acquisition time, a useful aFOV with image noise below 15% was only achievable using Gaussian filtering with axial extents of between 83 and 103 cm when going from 2 to 6 mm full-width-half-maximum, respectively.

**Conclusion:**

Image noise increases substantially towards the ends of the aFOV. However, good IQ in compliance with generally accepted benchmarks is achievable for an aFOV of > 90 cm. When accepting higher image noise or using dedicated protocol settings such as stronger filtering a useful aFOV of around 1 m can be achieved for a 2 min acquisition time.

**Supplementary Information:**

The online version contains supplementary material available at 10.1186/s40658-022-00516-5.

## Introduction

Since the first commercial installations at the beginning of this century, hybrid positron emission tomography and computed tomography (PET/CT) has been accepted as a standard-of-care imaging modality in oncology [1]. This clinical adoption has been accompanied by continuous performance and workflow improvements in the PET/CT systems due to ongoing technical innovation [2]. In recent years, significant development in PET technology helped to increase sensitivity with the use of extended axial field-of-views (aFOV). Volume sensitivity is defined as the number of detected true events per unit time for each unit of activity present within a source, which is mainly determined by the solid angle coverage of the annulus of detectors surrounding the patient. Commercially available PET/CT systems with aFOVs between 16 and 30 cm yield volume sensitivities in the range of 0.6–2% (or 6–20 kcps/MBq) [3]. To increase sensitivity, the axial volume coverage can be maximized by increasing the axial length of the PET system [4]. This approach has been followed already more than 15 years ago [5, 6], however, did not result in a commercially available system.

The substantial improvements made in detector technology and computational capabilities, invigorated the concept of extended aFOV PET and a total-body (TB) PET/CT system designed for non-human primate studies, the so-called mini-Explorer, was introduced in 2018 by UC Davis and Siemens Healthineers (Knoxville, TN, USA) offering an extended aFOV of 45.7 cm [7]. Shortly thereafter, the Explorer consortium (UC Davis and United Imaging Healthcare (Shanghai, China)) developed a clinical TB PET/CT scanner with 194 cm aFOV, the uExplorer [8, 9] and the University of Pennsylvania and Philips Healthcare (Cleveland, Ohio, USA) presented the PennPET Explorer, a PET/CT system designed for an axial length between 64 and 143 cm [10, 11]. This was followed in 2020 by the introduction of the Biograph Vision Quadra PET/CT system (“Quadra”) by Siemens Healthineers (Knoxville, TN, USA) with an aFOV of 106 cm offering anatomical coverage roughly from head to thighs [12, 13].

Due to the extended aFOV and sensitivity, this class of TB PET scanners will enable new fields of applications. With increased sensitivity, it is now possible to significantly reduce the amount of administered radiotracer activity resulting in a decrease of the patient and staff radiation dose. This may be beneficial for paediatric examinations [14] and may also potentially enable PET-based screening applications. The higher sensitivity can also be used to enable short time frames, which result in improved quality and temporal resolution in dynamic imaging and improved kinetic modelling. High sensitivity and anatomical coverage enables ultra-short acquisition times rendering breath hold PET acquisition approaches as feasible [2, 15]. Furthermore, TB PET will also be of notable interest for long half-life radioisotopes such as ^64^Cu or ^89^Zr, as enabler for immuno-imaging, where measurements up to multiple day post-injections are desired, e.g. to study ^89^Zr labelled antibody bio-distribution and pharmacokinetics [16]. In addition to the higher sensitivity, another important advantage of TB PET scanners is the possibility of simultaneous imaging of multiple organs. This fosters dynamic whole-body imaging, aiding a better understanding of tracer distribution and enables organ axis imaging [15, 17].

However, to assess if a TB PET system is suitable for applications, such as the simultaneous examination of large anatomical regions, the nominal total sensitivity as for example measured according to the National Electrical Manufacturers Association (NEMA) NU 2 protocols [18] is not a sufficient metric. The reason is that the determination of the total sensitivity is based on the cumulative count rate over all transaxial slices for a line source with an axial extend of only 70 cm. For a more meaningful assessment of the scanner’s sensitivity, the sensitivity per imaging plane needs to be taken into consideration as this will determine the noise in the reconstructed image volume at the respective axial position. With the current design of all PET systems based on a cylindrical detector arrangement, the sensitivity per slice always decreases towards the end of the aFOV. This decrease of sensitivity is compensated by overlapping bed positions in multi-bed examinations or continuous-bed-motion (CBM) techniques to cover extended areas [19]. In the case of TB PET, multi-bed examinations are no longer intended for a variety of applications [15, 17], hence the sensitivity reduction at the axial ends must be considered when extended areas are investigated.

Therefore, the aim of this study is to evaluate the image quality (IQ) and noise properties along the 106 cm aFOV of the Quadra TB PET/CT system based on phantom examinations and to estimate the clinical useful aFOV extend for single-bed PET imaging with [^18^F]-based tracers.

## Material and methods

### Quadra PET/CT system

All measurements were taken using a Quadra PET/CT system (Siemens Healthineers, Knoxville, TN, USA) installed at the University hospital in Tübingen, Germany. The system is based on 3.2 × 3.2 × 20 mm lutetium oxyorthosilicate (LSO) crystals coupled to silicon photomultipliers (SiPMs) offering a time-of-flight (TOF) resolution of typically 228 ps [12]. The detectors are cylindrically arranged with a diameter of 82 cm and an axial length of 106 cm [12]. Acquisitions were performed using the scanner software version VR10D using a maximum ring difference (MRD) of 85. Of note, to take all line of responses into account a MRD of 322 would be necessary, which will be available with a future software release. With the MRD 85 settings the total sensitivity of the system according to NEMA NU 2-2018 is 83 kcps/MBq with a constant sensitivity profile of around 200 cps/MBq/plane across the central aFOV [12].

### Phantom measurements

A standard NEMA IQ phantom was used [18]. The phantom was placed on the patient bed with the centre of the lung insert aligned with the transaxial centre of the FOV and the centres of the spheres aligned within the same transaxial plane. The experiments were performed using [^18^F]FDG with three different sphere-to-background-ratios (SBR) of 8:1, 4:1 and 2:1 (Table 1). For each SBR four measurements of 10 min each were taken as dynamic acquisition at four different axial positions. The centres of the spheres were positioned in the axial centre of the system (POS-0) and with distances of 250 mm (POS-250), 450 mm (POS-450) and 505 mm (POS-505) from POS-0 towards the end of the aFOV (the end of the aFOV corresponds to 530 mm distance to POS-0). At POS-505 the background and lung insert of the phantom are covering the slice corresponding to the edge of the aFOV.

**Table 1:** Activity concentrations and SBRs for all phantom acquisitions as determined with the dose calibrator

SBR (calculated)	Axial position (mm)	Sphere activity concentration (kBq/ml)	Background activity concentration (kBq/ml)	Fraction of rebinned acquisition time used for reconstruction
8:1 (8.0:1)	0	26.02	3.26	0.77
	250	23.66	2.97	0.84
	450	21.66	2.72	0.92
	505	19.95	2.50	1.00
4:1 (3.9:1)	0	12.99	3.30	0.76
	250	11.81	3.00	0.84
	450	10.88	2.76	0.91
	505	9.90	2.51	1.00
2:1 (2.0:1)	0	6.48	3.29	0.78
	250	6.01	3.05	0.84
	450	5.47	2.77	0.92
	505	5.04	2.56	1.00

The phantom background was filled with an activity concentration of approximately 2.5 kBq/ml at the start of the last measurement (POS-505) equivalent to a standard-of-care [^18^F]FDG examination after injection of ~ 250 MBq (6.8 mCi) [^18^F]FDG for a 70 kg person and performing the imaging 60 min post-injection.

The dynamic PET data were rebinned to simulate a 600 s, 120 s, 60 s, 30 s and 15 s total acquisition. To account for the higher activity present in the phantom during the scans at POS-0 to POS-450 the rebinned acquisition time for reconstruction was shortened accordingly (Table 1).

Image reconstruction was performed following a standard clinical protocol using an Ordinary Poisson Ordered Subsets Expectation Maximisation (OP-OSEM) algorithm with point-spread-function (PSF) modelling and using TOF information. A matrix size of 440 × 440 × 645 was used resulting in a 1.65 × 1.65 × 1.65 mm^3^ isotropic voxel size. Attenuation correction was performed based on a standard-of-care diagnostic CT scan (120 kVp tube potential, automatic tube current modulation with 210 mAs ref.) acquired prior to the emission measurements. All images were evaluated without use of a post-reconstruction filter to have reference to evaluate the impact of different filter sizes on noise and contrast. In addition, to meet standard-of-care reconstruction protocols, post-reconstruction 3D Gaussian filtering of 2, 3, 4, 5 and 6 mm full width at half maximum (FWHM) was applied to the 120 s, 60 s, 30 s and 15 s acquisitions to evaluate the background noise.

### Evaluation

Reconstructed image data were analysed using AMIDE [20]. Calculations were performed using MATLAB 2021a (MathWorks Inc., Natick, USA) and the software R v.3.3.1 (R Foundation for Statistical computing, Vienna, Austria).

#### Image noise

**Fig. 1:**
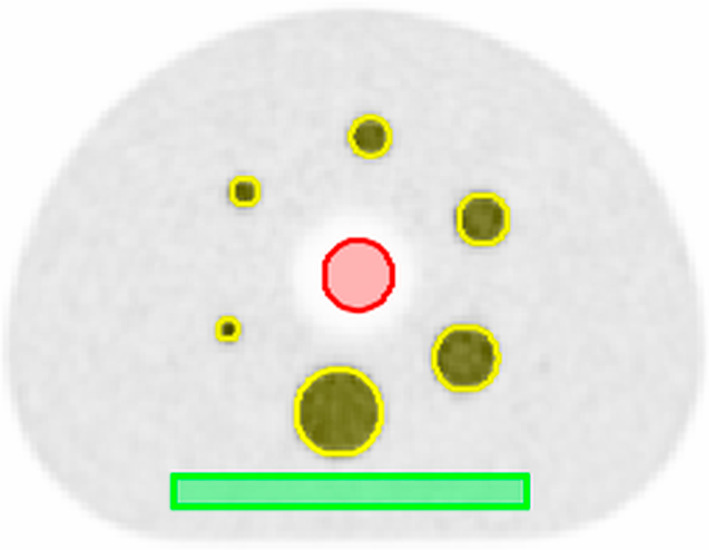
Central slice of a 600 s acquisition with 8:1 SBR of the IQ phantom. The VOIs of the background (green), spheres (yellow) and lung (red) are used to extract the voxel data for contrast and noise calculations

The evaluation of image noise and accuracy of correction methods was performed for the phantom with 8:1 SBR. To evaluate the noise properties across the aFOV, a box-shaped volume-of-interest (VOI) with in-plane dimensions of 150 × 15 mm^2^ and an axial dimension of 170 mm covering almost the total length of the phantom was placed in the background region (Fig. 1). A short margin towards the end of the background compartment was excluded to avoid potential partial volume effects or artefacts at the border between background compartment and phantom housing. Voxel data were extracted from the background VOI and the axial position-dependent noise was calculated as coefficient-of-variation (CV) by dividing the standard deviation (SD) by the mean voxel values for each transaxial slice. A fit modelling the Poisson noise according to the equation

1 $${\text{CV}}_{{{\text{fit}}}} \left( z \right) = \frac{1}{{\sqrt {c \times \left( {z + a} \right)} }}$$

was applied to the axial position-dependent noise for the measurement at POS-505. CV_fit_ is the fitted CV value and *a* accounts for the shift of the fit function in z direction (to achieve a zero value at the *z* position directly outside the aFOV). The fit parameter *c* models the proportionality between axial position and number of events (can be interpreted as the slope of the sensitivity profile). The fit is used to determine at which axial position the CV exceeds the set threshold for clinical acceptable noise of 15% as defined by [21–24]. The useful aFOV in this manuscript is defined as the axial extend for which this threshold is not exceeded.

Furthermore, a cylindrical VOI with 30 mm diameter and an axial dimension of 170 mm was placed in the lung insert (Fig. 1) to determine the accuracy of the scatter, randoms and attenuation correction. The mean voxel value of each transaxial slice of the lung VOI was divided by the mean voxel value of the total background VOI to obtain the axial position-dependent lung residual error. In addition, the average lung residual error is calculated as the mean of the lung residual errors over the four measurement positions.

#### Image quality

IQ was assessed by means of image contrast metrics. Six spherical VOIs with dimensions of the original sphere sizes of 37 mm, 28 mm, 22 mm, 17 mm, 13 mm and 10 mm (inner diameter) were placed over the spheres in the phantom (Fig. 1). Contrasts for all axial positions and SBRs were calculated for each sphere by dividing the mean voxel value for each sphere with the mean voxel value from the total background compartment and the calculated SBR.

#### Patient data

To illustrate the reachable coverage within the defined useful aFOV as found for the phantom experiments, a [^18^F]FDG PET/CT scan of a female patient suffering from malignant melanoma was added to this evaluation. A 10 min PET scan was acquired 111 min p. i. of 245 MBq [^18^F]FDG on the Quadra system. Low dose CT was used for attenuation correction (100 kVp tube potential, automatic tube current modulation with 17 mAs ref). PET data were rebinned to 190 s which is equivalent to the 120 s acquisition of the phantom measurements and the same reconstruction parameters as for the phantom studies were used. To cross-check the validity of the phantom experiments to predict noise within the patient, the noise within the liver was compared to the noise found in the phantom experiments. Therefore, a spherical VOI with a diameter of 4 cm inside the liver was used to determine image noise calculated as CV by dividing the SD by the mean of the voxel values.

The study involving human participants were conducted in accordance with the principles embodied in the Declaration of Helsinki and were reviewed and approved by the Ethics Committee at the Faculty of Medicine, University of Tübingen (Nr. 773/2021BO2). Written informed consent was obtained from the patient.

## Results

### Image noise

**Fig. 2:**
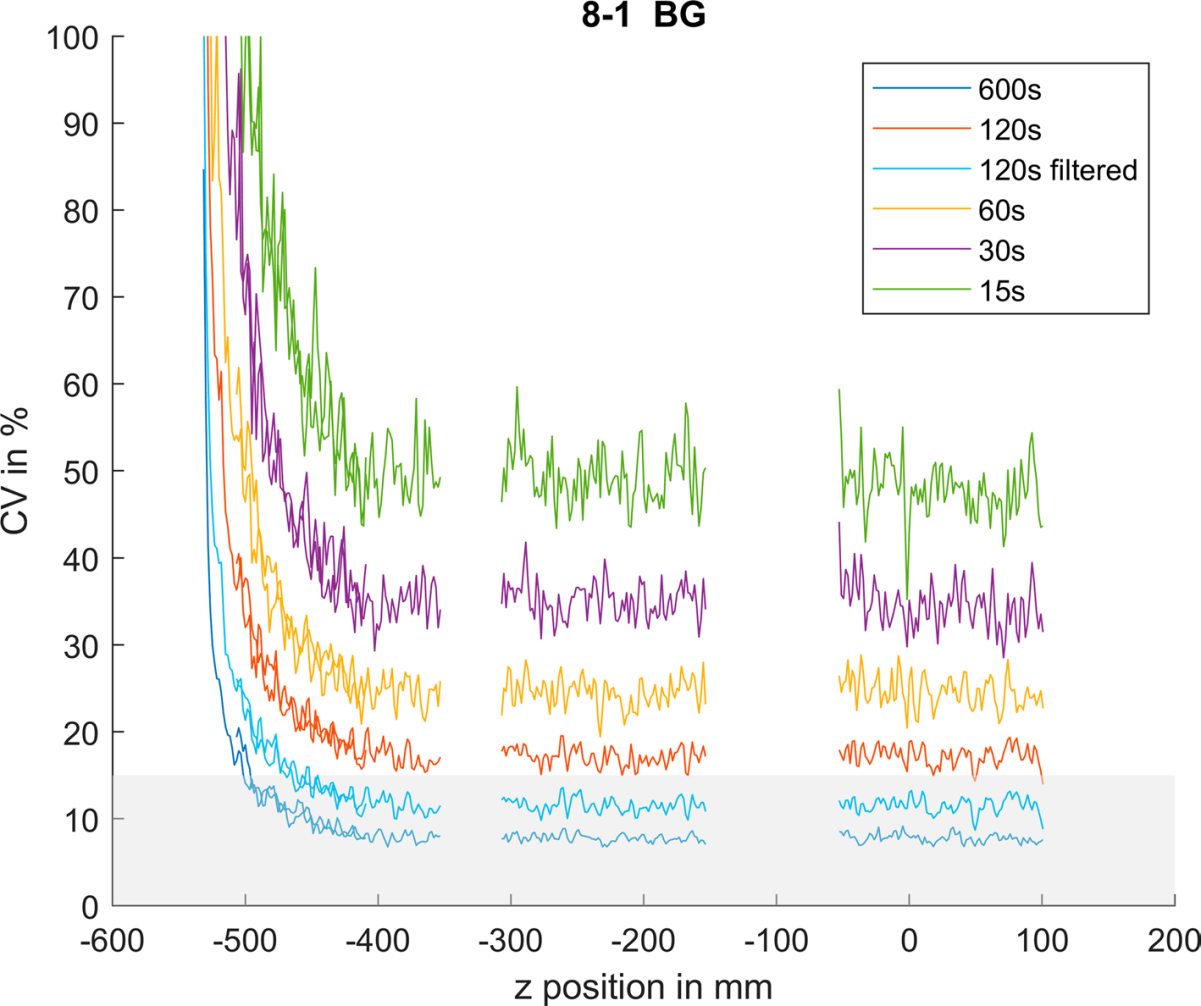
Image noise expressed as CVs [%] for the different acquisition times and axial imaging positions including an example of a filtered data set (120 s with 3 mm FWHM Gaussian filter)

Image noise was constant from the centre to an axial offset of 400 mm with an average CV of 7.9%, 17.3%, 24.6%, 35.2% and 49.3% for the 600 s, 120 s, 60 s, 30 s and 15 s acquisitions, respectively (Fig. 2). Applying a Gaussian post-reconstruction filter to the acquisition ≤ 120 s reduced the noise depending on the used FWHM of the Gaussian kernel (Table 2). At axial offsets from the centre above 400 mm, image noise increased following the function of the Poisson noise (Eq. 1) (Fig. 2). The increase in noise was in particular prominent for the 30 s and 15 s measurements. The longer measurements revealed a lower increase in noise due to the improved count statistics.

**Table 2:** Background CV (%) in the central position and useful axial FOV with background CV < 15% for the reconstructions after applying different Gaussian filter kernels

Gaussian filter FWHM (mm)	Equivalent acquisition time (s)	Background CV (%)	aFOV with CV < 15% (cm)
0	600	7.9	99.8
0	120	17.3	NA
2	120	14.6	83.0
3	120	12.5	91.6
4	120	9.3	97.0
5	120	7.5	100.4
6	120	6.1	102.8
0	60	24.6	NA
2	60	20.7	NA
3	60	16.6	NA
4	60	13.3	88.5
6	60	8.7	95.1
0	30	35.2	NA
2	30	28.8	NA
3	30	22.8	NA
4	30	18.1	NA
5	30	14.0	81.8
6	30	11.2	90.5
0	15	49.3	NA
2	15	39.9	NA
3	15	31.3	NA
4	15	24.7	NA
5	15	19.5	NA
6	15	15.6	NA

NA denotes settings where a CV < 15% could not be reached at any position throughout the aFOV

**Fig. 3:**
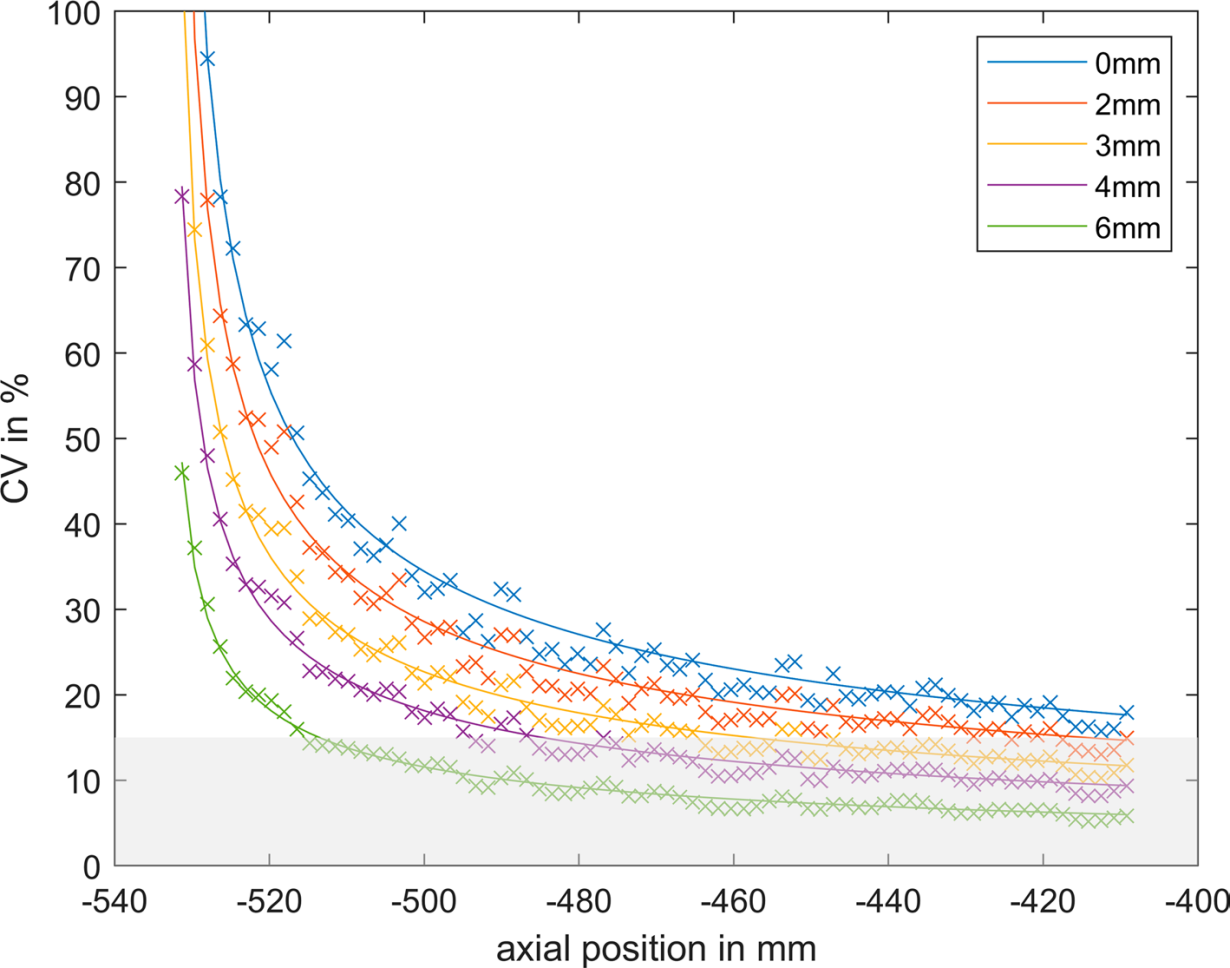
Example of the fit functions of axial dependence of CV [%] at POS-505 for 120 s acquisition with different Gaussian filters. The legend shows the FWHM of the Gaussian filter. The grey area shows the area < 15% background noise

For the 600 s measurement, the CV threshold of 15% was exceeded at an axial offset of 500 mm from the centre (Fig. 3). For the shorter acquisitions, the 15% threshold was always exceeded when no post-filtering was applied. After applying a 3 mm FWHM Gaussian filter to the 120 s measurements, the 15% threshold was exceeded at 458 mm axial offset determined by the fit function (Eq. 1) (Fig. 3).

### Lung residual error

**Fig. 4:**
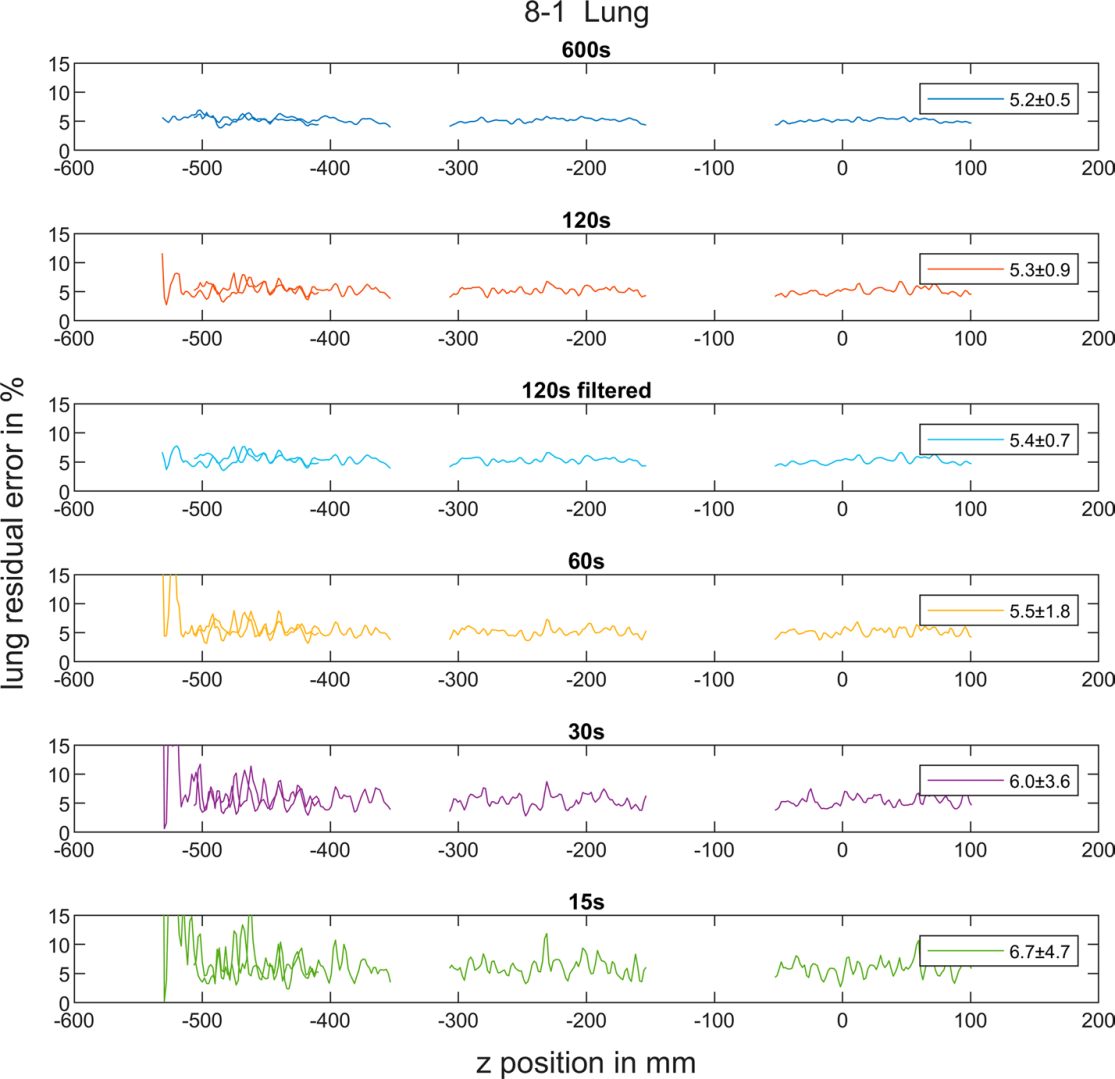
Axial variation of the lung residual error [%] including an example of a filtered data set (120 s with 3 mm FWHM Gaussian filter). The mean and SD of the lung residual error are given in the legend of the respective plots

The average lung residual error was determined to be 5.2 ± 0.5%, 5.3 ± 0.9%, 5.5 ± 1.8%, 6.0 ± 3.6% and 6.7 ± 4.7% for an acquisition of 600 s, 120 s, 60 s, 30 s and 15 s, respectively. Applying a Gaussian filter revealed no significant impact on the lung residual error compared to the unfiltered data. The lung residual error showed the lowest variation (10% relative SD) over the entire aFOV for the 600 s acquisition and was still in an acceptable range for the 120 s (17% relative SD). For the 60 s, 30 s and 15 s acquisition the error increased significantly (33%, 60% and 70% relative SD, respectively). At axial offsets from the centre above 400 mm the lung residual error significantly increased for the 30 s and 15 s acquisition (Fig. 4).

### Image quality

**Fig. 5:**
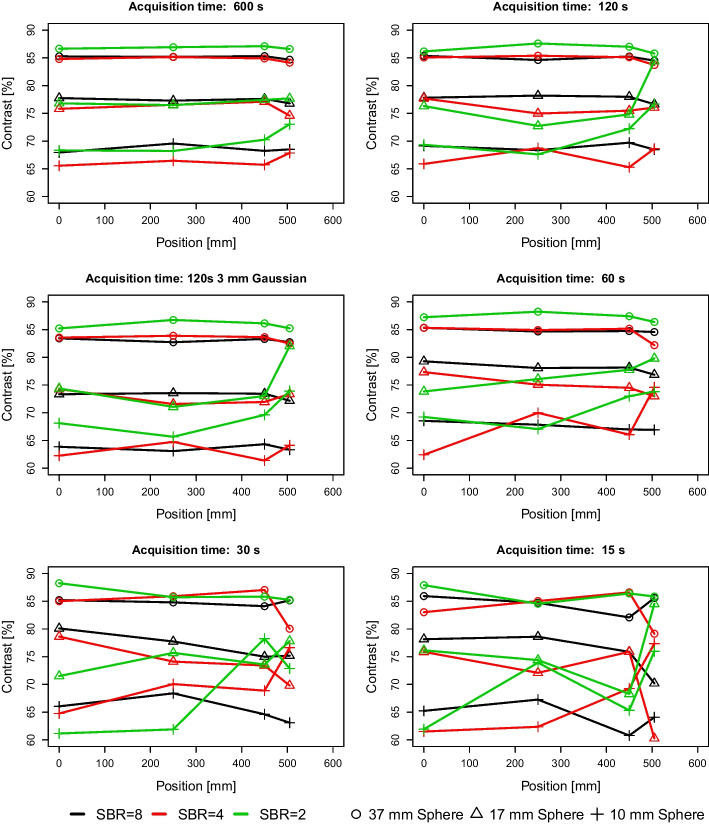
Image contrasts for three representative sphere sizes for the different SBRs and axial positions

The IQ assessment at the central position (POS-0) using 600 s acquisition time and an SBR of 8:1 resulted in contrasts between 85 and 61% for the different sphere sizes (Table 3). These values are comparable to contrasts published in the literature for another Quadra and a Biograph Vision 600 PET/CT system [12, 25]. For the 600 s and 120 s measurements, contrasts were comparable for SBRs of 8:1 and 4:1 and for sphere sizes larger than 17 mm for SBR 2:1 (Table 3 and Fig. 5). For acquisition times of 30 s and 15 s, the variability of the contrasts increased, in particular for the smaller spheres and lower SBRs. All calculated contrasts for the different acquisition times and positions can be found in Additional File 1: Table S1.

**Table 3:** Image contrasts at the central position (POS-0) for the different sphere sizes, SBRs and acquisition times. Of note, the slightly higher contrast for the 22 mm sphere size than found for the 28 mm sphere size is expected to be related to Gibbs artefacts well known in PSF reconstructions, in particular evident in unfiltered reconstructions [32]

	SBR = 8:1						SBR = 4:1						SBR = 2:1					
Acquisition time	600 s	120 s	120 s filtered	60 s	30 s	15 s	600 s	120 s	120 s filtered	60 s	30 s	15 s	600 s	120 s	120 s filtered	60 s	30 s	15 s
*Sphere size (mm)*																		
37	85.3	85.4	83.4	85.3	85.2	85.9	84.8	85.1	83.6	85.3	85.0	83	86.7	86.2	85.2	87.2	88.3	87.9
28	79.9	79.4	76.9	78.6	78.1	77.6	79.4	79.1	77.3	79.2	79.8	76.0	79.5	79.8	78.7	81.0	78.3	80.6
22	81.5	81.8	78.2	82.1	83.0	82.4	80.8	80.3	77.8	80.9	80.7	78.5	81.4	80.7	79.3	78.8	76.3	77.2
17	77.8	77.8	73.3	79.3	80.1	78.2	75.8	77.7	74.3	77.3	78.6	75.9	76.8	76.3	74.4	73.8	71.5	76.1
13	67.9	69.1	63.9	68.5	66.0	65.2	65.6	65.9	62.3	62.4	64.8	61.5	68.3	69.3	68.1	69.2	61.2	61.9
10	61.1	60.4	54.2	59.3	62.6	53.4	59.6	59.3	54.7	55.7	56.4	55.4	67.1	68.1	65.2	70.2	70.7	72.5

Comparing the contrast recovery along the aFOV revealed a stable behaviour from the centre of the FOV (POS-0) to POS-450 for the 600 s and 120 s acquisition time (Fig. 5) and spheres with diameters larger than 13 mm for SBRs 8:1 and 4:1. The smallest SBR of 2:1 revealed larger deviations. Furthermore, for the edge position POS-505 the variability in contrasts compared to other positions increased for all investigated SBRs. This was in particular evident for the small spheres. Contrast ratios determined for acquisition times of 30 s and 15 s showed a strong fluctuating behaviour at position POS-505 (Fig. 5).

**Fig. 6:**
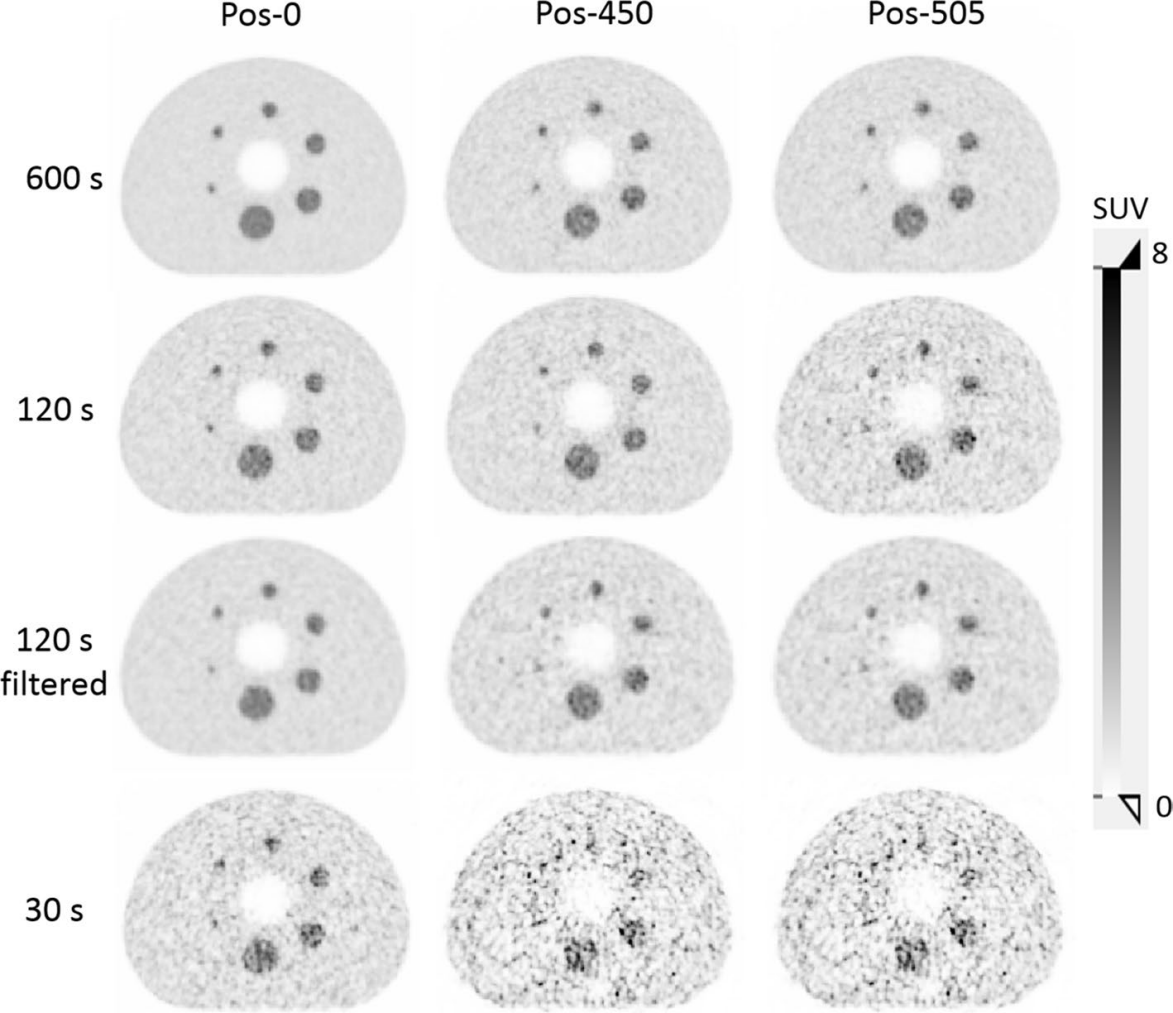
Central slice of the IQ phantom measurements with a SBR of 4:1 at different axial positions for the different acquisition durations. Further, representations including also the 15 s data can be found in the Additional File 2 figures

These findings were in-line with the visual assessment of the IQ that demonstrated substandard quality at all positions for 15 s and 30 s acquisition time and at Pos-505 for the 120 s acquisition (Fig. 6, Additional File 2: Figs. S1, S2 and S3). The filtered acquisition of 120 s revealed similar contrast values as to the unfiltered one (Fig. 5), although the qualitative assessment of the reconstructed image shows reduced noise level, as a result of the Gaussian filter (Fig. 6).

### Patient example

**Fig. 7:**
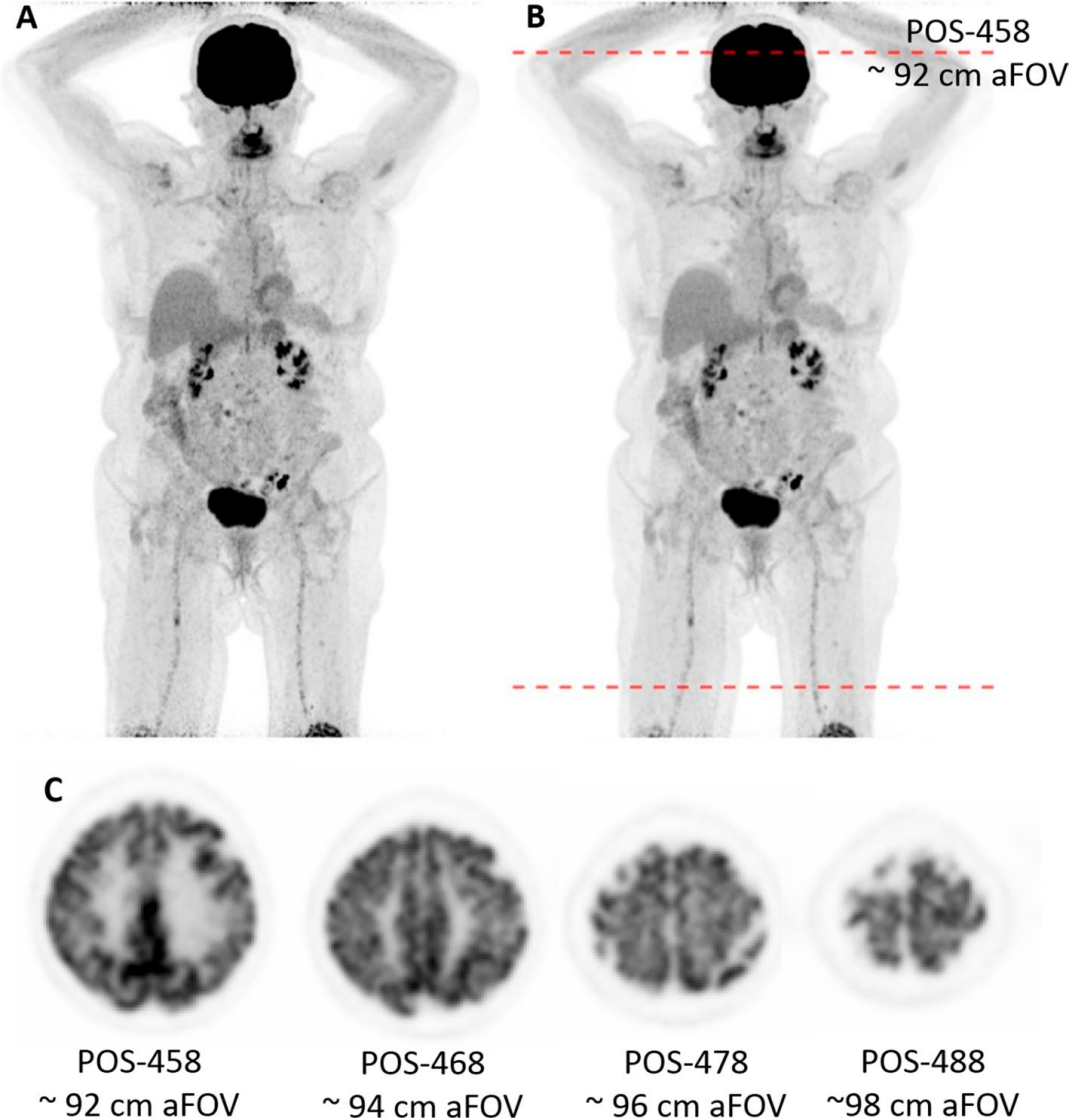
**A** and **B** Maximum intensity projection of a patient suffering from melanoma with an equivalent scan duration of 120 s without (**A**) and with a 3 mm Gaussian filter (**B**) applied. The lines indicate the extent of the useful aFOV of 92 cm. The increase in image noise towards the end of the aFOV is clearly visible in both reconstructions. **C** Axial slices through the brain for the 120 s acquisition with 3 mm Gaussian filter at the calculated edge of the useful aFOV (POS-458) and at three additional positions towards the end of the aFOV still showing clinical sufficient IQ for the brain

The noise in the patient images visually increased towards the ends of the aFOV (Fig. 7). After applying a 3 mm Gaussian filter, the noise was reduced with no clinically significative decrease of image details. This is in accordance with a CV inside the liver of 13.6% and 9.7% without and with a 3 mm filter, respectively. According to the evaluation of the phantom data the useful aFOV is 92 cm for the patient scan with a 3 mm filter. However, by clinical standards, an image with (qualitatively) acceptable IQ was observed up to approximately 98 cm aFOV (Fig. 7).

## Discussion

This study evaluates the IQ along the 106 cm long aFOV of the Quadra TB PET/CT system and determines the useful aFOV for clinical applications. To define useful aFOV of the Quadra PET/CT system, a standard [^18^F]FDG examination for tumour imaging was selected as a benchmark example, assuming a PET scan 60 min post-injection of a typical dose of 250 MBq (6.8 mCi) for a 70 kg patient [26, 27]. As metric for acceptable IQ a 15% background CV in the NEMA IQ phantom was used as recommended by the European Association of Nuclear Medicine (EANM), their associated Research GmbH (EARL) initiative [21] and the European Federation of Medical Physics (EFOMP) [22]. Using these benchmarks, the useful aFOV was assessed to be 92 cm in the Quadra system for a typical clinical reconstruction protocol (Gaussian filter of 3 mm) with a 2 min acquisition time (Tables 2 and 3). This aFOV is sufficient to cover an area including the head and the trunk for most subjects, and thus, allows a simultaneous examination of all major organs (Fig. 7).

The linearly decreasing sensitivity towards the end of the aFOV has a substantial impact on the IQ, and thus, limits the useful aFOV for single-bed acquisition protocols. The image noise is well characterized by the Poisson noise (Fig. 3). This was expected as a reduced sensitivity is directly coupled to increased noise based on the fundamental statistical properties of counting experiments as performed in PET [28].

However, the exact extent of the useful aFOV depends not only on the sensitivity profile of the system but also on the desired acceptable IQ. Thus, the useful aFOV is determined by a combination of the target frame duration for imaging, the applicable radiotracer dose, the desired spatial resolution and the acceptable noise. With a definition of a maximum noise level as main IQ benchmark, the use of longer frame durations or higher injected activities allows to simultaneously cover extended axial areas. Further, the useful aFOV can be increased by the selection of proper reconstruction protocols aimed to reducing the noise, such as the use of lower numbers of iterations [29], larger voxel sizes [30] or post-reconstruction filtering as done in this study (Table 2). However, such adjustments of the reconstruction protocols might also be associated with a reduced image resolution. This needs to be considered when such adjustments are performed. Of note, in this study we evaluated only standard reconstruction settings including PSF correction. The effect of PSF on image quality is well described [31–33] in the literature. PSF correction is known to reduce background noise and to enhance contrast [32]. Avoiding PSF correction is expected to reduce the useful aFOV as defined in this study due to increased noise and a reduction of image contrast.

Nevertheless, in reality, increased noise at the axial ends of the examination areas is well known from standard PET/CT examination [34] and noise levels above a CV of 15% as defined by the EANM or EFOMP [21, 22] are often accepted in routine operation. Furthermore, the local image noise in tissues of interest with high tracer uptake is, in general, lower as assessed using the CV in the background of the NEMA IQ phantom. This behaviour is exemplified by the noise assessed in the liver of the patient examination yielding a liver CV of 11.8% and 8.3% compared to a CV of 17.3% and 12.5% in the respective phantom scan for the unfiltered and filtered images, respectively. This behaviour can easily be explained by the locally higher counting statistic in such high uptake organs. Comparing the theoretical difference in noise when assuming a 1/√*n* behaviour between an SUV of 2.4 as measured in the liver compared to the activity in the background compartment of the phantom (SUV = 1) reveals a reduction of 35% in image noise. This compares well with the actual difference found in this study. Therefore, tracers with a high brain uptake as it is the case for [^18^F]FDG might allow to achieve a sufficient IQ within this organs also slightly outside the useful aFOV as defined within this study (Fig. 7).

In general, examination areas of above one metre in a single-bed position protocol are practically achievable in the Quadra system, when accepting image noise in a reasonable range above 15% CV distal of the aFOV or with adjusted acquisition and/or reconstruction protocols. Only for very short acquisition times (< 30 s) it seems unrealistic that a clinical sufficient IQ can be reached for an area far beyond the central 80 cm aFOV without substantial post-filtering of the images (Table 2), and thus, a respective degradation of resolution.

Of note, the current version of the Quadra uses an MRD of 85 for image reconstruction. Therefore, the evaluation in this study was done using the MRD of 85 as the only currently clinically available acquisition mode. The MRD 322 settings were not investigated as at the time of the study available external reconstruction tools may not reflect the performance of the clinical reconstruction software expected to be implemented in a future software update. The anticipated extension of the MRD to 322 (using all possible lines-of-response) is expected to have significant effect on the sensitivity profile, and thus, the noise properties; MRD322 will provide a triangle shaped sensitivity profile with a 2.7-fold increased maximal sensitivity in the axial centre [12]. This will enable improved noise properties in particular in the centre of the aFOV. Here, it is expected that it will be possible to reach areas < 15% CV also for short acquisition times < 60 s. However, the triangle shaped sensitivity profile will also lead to a continuous change in noise properties along the aFOV. This needs to be considered in the set-up and optimisation of examination protocols.

## Conclusion

The decreasing sensitivity towards the ends of the aFOV has a substantial impact on IQ in the axial end regions of the Quadra PET/CT system. The useful aFOV was assessed to be 92 cm for a typical clinical reconstruction protocol with 2 min acquisition time and generally accepted IQ benchmarks. However, with adjusted acquisition and/or reconstruction protocols such as the use of Gaussian filtering or when accepting higher image noise towards the ends of the aFOV, examination areas of above one metre are realistically achievable.

## Supplementary Information

**Additional file1**. **Table S1**. Calculated contrasts for all sphere sizes, axial positions and different acquisition times.

**Additional file2**. **Figure S1**: Central slice of the NEMA IQ phantom for the Pos-0 (centre of the spheres placed at the centre of the axial FOV). Data are presented for the different SBRs (8 to 1, 4 to 1 and 2 to 1) and for the different acquisition times of 600 s, 120 s, 60 s, 30 s and 15 s. In addition the centre slice of the 120 s acquisition is given after applying a 3 mm FWHM Gaussian filtering. **Figure S2**: Central slice of the NEMA IQ phantom for the Pos-450 (centre of the spheres placed at 450 mm offset of the centre of the axial FOV). Data are presented for the different SBRs (8 to 1, 4 to 1 and 2 to 1) and for the different acquisition times of 600 s, 120 s, 60 s, 30 s and 15 s. In addition the centre slice of the 120 s acquisition is given after applying a 3 mm FWHM Gaussian filtering. **Figure S3**: Central slice of the NEMA IQ phantom for the Pos-505 (centre of the spheres placed at 505 mm offset of the centre of the axial FOV). Data are presented for the different SBRs (8 to 1, 4 to 1 and 2 to 1) and for the different acquisition times of 600 s, 120 s, 60 s, 30 s and 15 s. In addition the centre slice of the 120 s acquisition is given after applying a 3 mm FWHM Gaussian filtering.

## Data Availability

Phantom data is available upon reasonable request from the corresponding authors.
