# Supplementary material for: Image quality assessment along the one metre axial field-of-view of the total-body Biograph Vision Quadra PET/CT system for 18F-FDG
Source: EJNMMI Phys. 2022 Dec 14;9:87. doi: 10.1186/s40658-022-00516-5 (PMC9747988; doi:10.1186/s40658-022-00516-5)
Supplement: Supplementary file 2 — Additional file2. Figure S1: Central slice of the NEMA IQ phantom for the Pos-0 (centre of the spheres placed at the centre of the axial FOV). Data are presented for the different SBRs (8 to 1, 4 to 1 and 2 to 1) and for the different acquisition times of 600 s, 120 s, 60 s, 30 s and 15 s. In addition the centre slice of the 120 s acquisition is given after applying a 3 mm FWHM Gaussian filtering. Figure S2: Central slice of the NEMA IQ phantom for the Pos-450 (centre of the spheres placed at 450 mm offset of the centre of the axial FOV). Data are presented for the different SBRs (8 to 1, 4 to 1 and 2 to 1) and for the different acquisition times of 600 s, 120 s, 60 s, 30 s and 15 s. In addition the centre slice of the 120 s acquisition is given after applying a 3 mm FWHM Gaussian filtering. Figure S3: Central slice of the NEMA IQ phantom for the Pos-505 (centre of the spheres placed at 505 mm offset of the centre of the axial FOV). Data are presented for the different SBRs (8 to 1, 4 to 1 and 2 to 1) and for the different acquisition times of 600 s, 120 s, 60 s, 30 s and 15 s. In addition the centre slice of the 120 s acquisition is given after applying a 3 mm FWHM Gaussian filtering. [file 40658_2022_516_MOESM2_ESM.pdf]

Center Position (Pos-0)

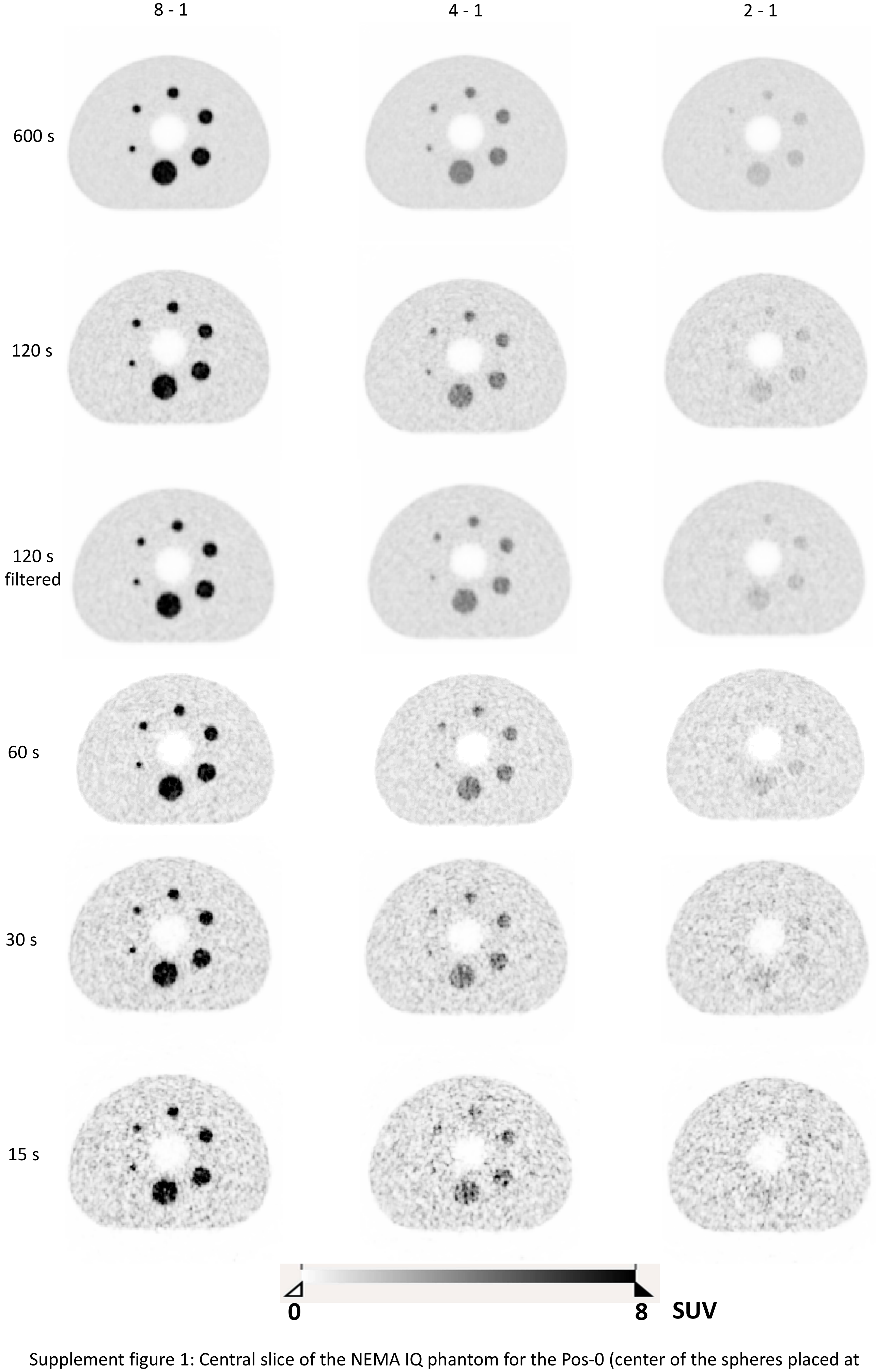

Supplement figure 1: Central slice of the NEMA IQ phantom for the Pos-0 (center of the spheres placed at the center of the axial FOV). Data are presented for the different SBRs (8 to 1, 4 to 1 and 2 to 1) and for the different acquisition times of 600 s, 120 s, 60 s, 30 s and 15 s. In addition the center slice of the 120 s acquisition is given after applying a 3 mm FWHM Gaussian filtering.

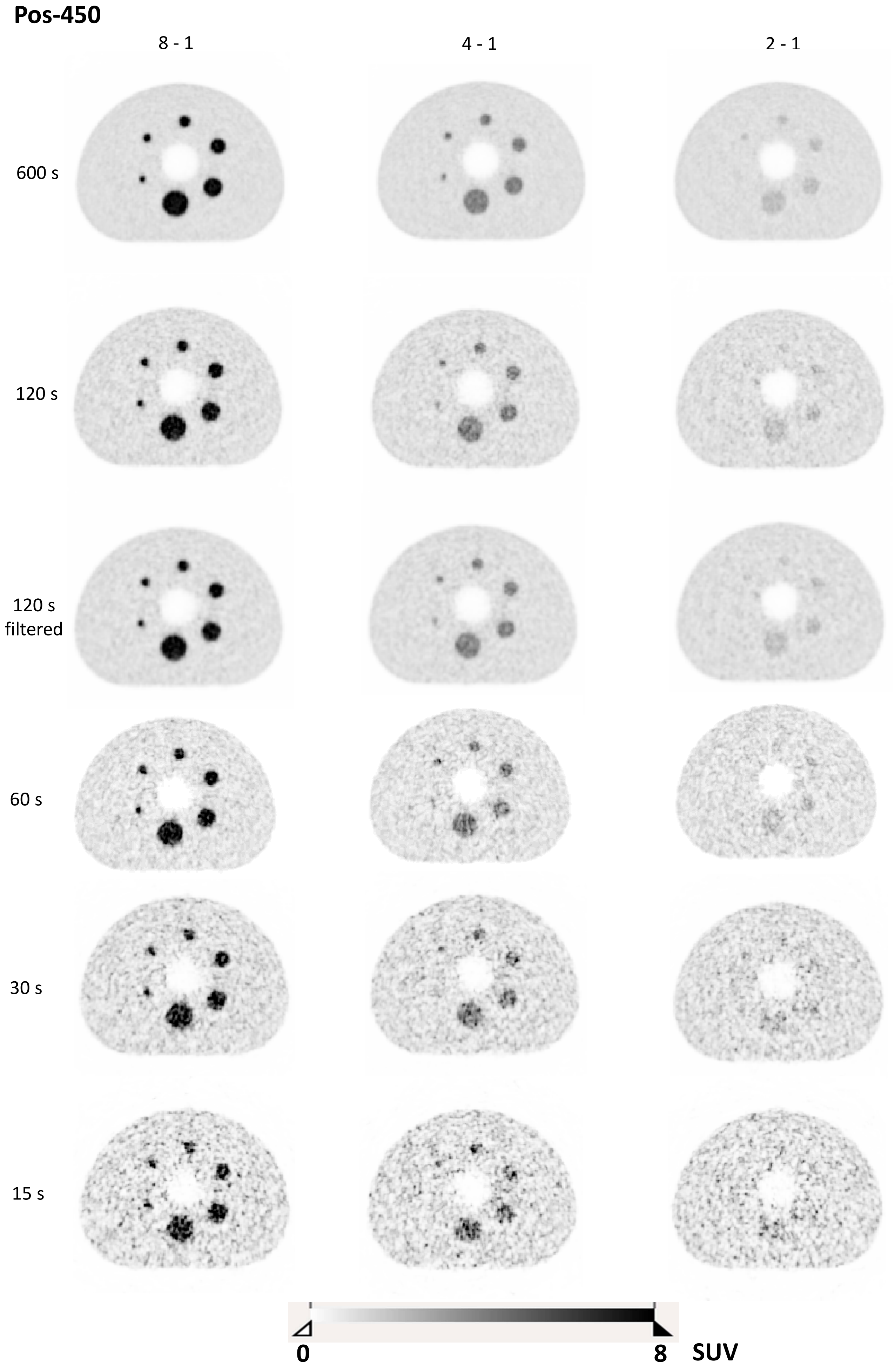

Supplement figure 2: Central slice of the NEMA IQ phantom for the Pos-450 (center of the spheres placed at 450 mm offset of the center of the axial FOV). Data are presented for the different SBRs (8 to 1, 4 to 1 and 2 to 1) and for the different acquisition times of 600 s, 120 s, 60 s, 30 s and 15 s. In addition the center slice of the 120 s acquisition is given after applying a 3 mm FWHM Gaussian filtering.

Pos-505

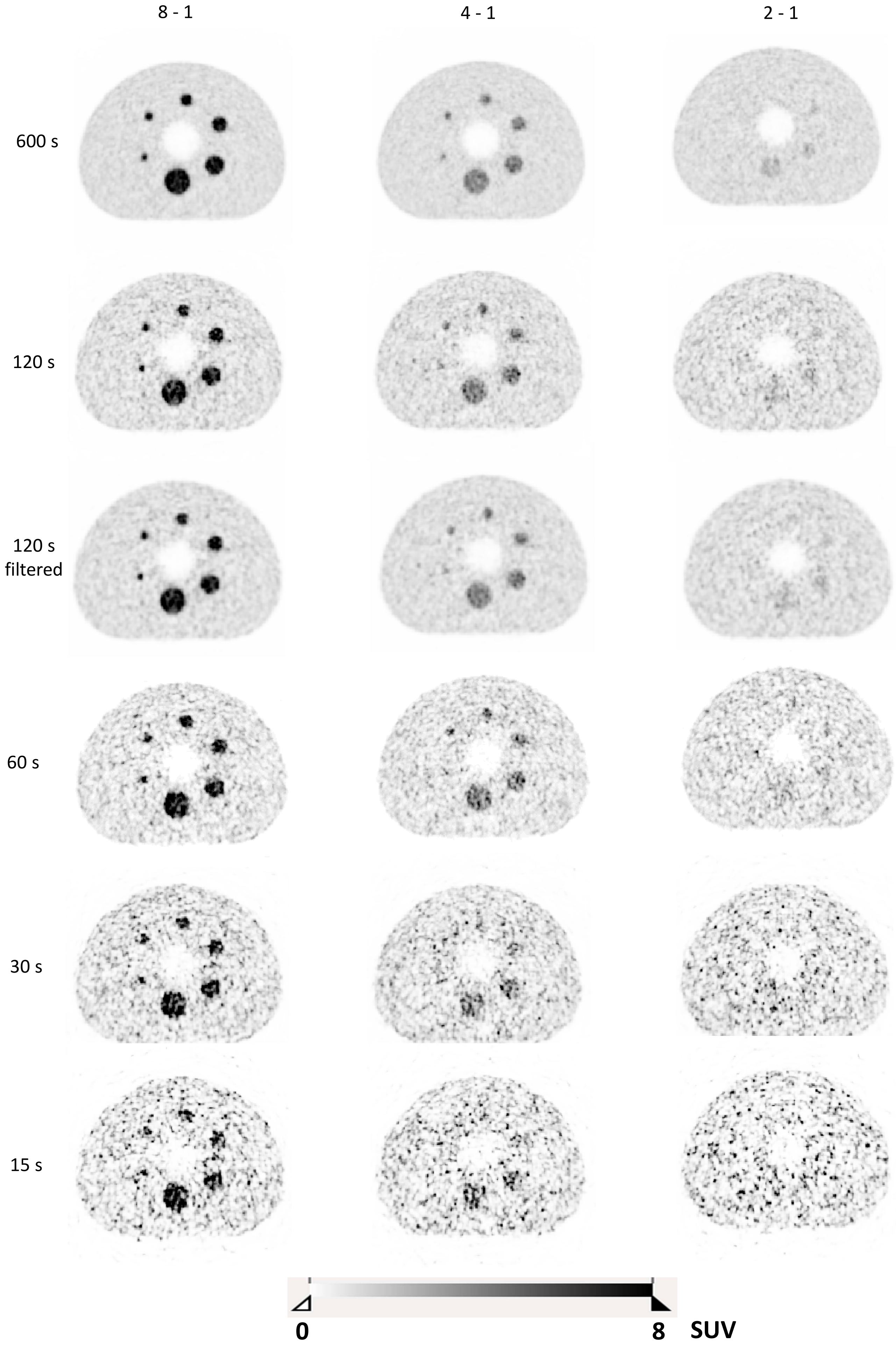

Supplement figure 3: Central slice of the NEMA IQ phantom for the Pos-505 (center of the spheres placed at 505 mm offset of the center of the axial FOV). Data are presented for the different SBRs (8 to 1, 4 to 1 and 2 to 1) and for the different acquisition times of 600 s, 120 s, 60 s, 30 s and 15 s. In addition the center slice of the 120 s acquisition is given after applying a 3 mm FWHM Gaussian filtering.
